# Supplementary material for: Altered effective connectivity in sensorimotor cortices is a signature of severity and clinical course in depression
Source: Proc Natl Acad Sci U S A. 2021 Sep 30;118(40):e2105730118. doi: 10.1073/pnas.2105730118 (PMC8501855; doi:10.1073/pnas.2105730118)
Supplement: Supplementary File [file pnas.2105730118.sapp.pdf]

Group Mean Effective Connectivity

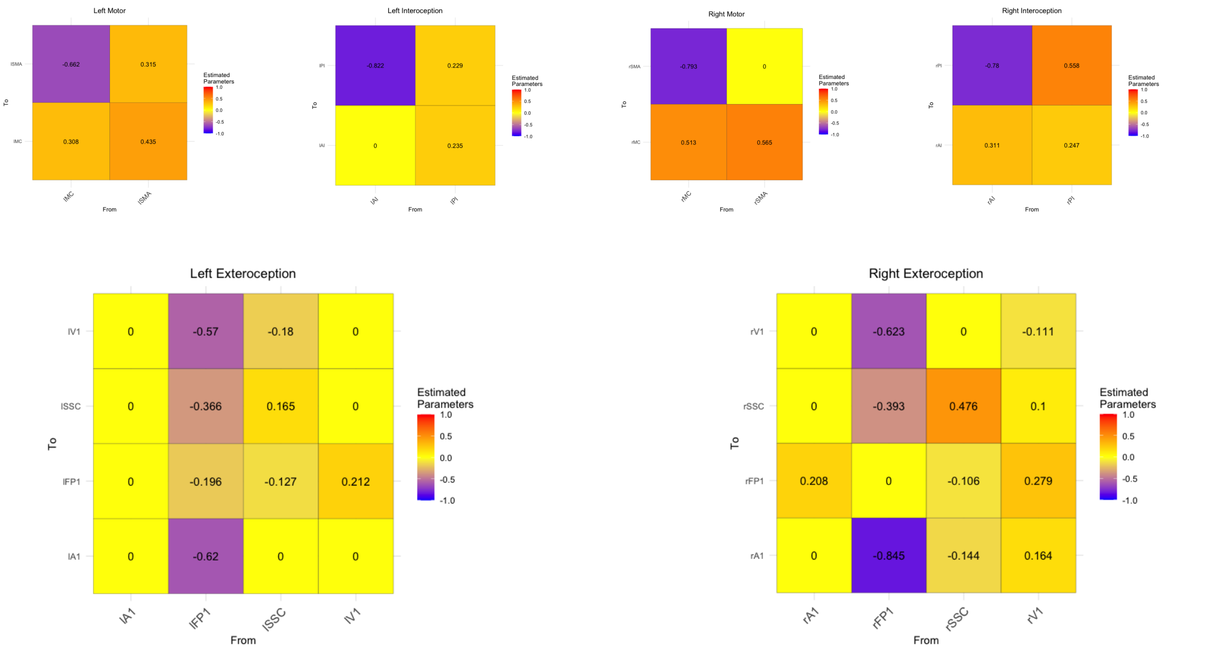

Changes in effective connectivity with BDI scores

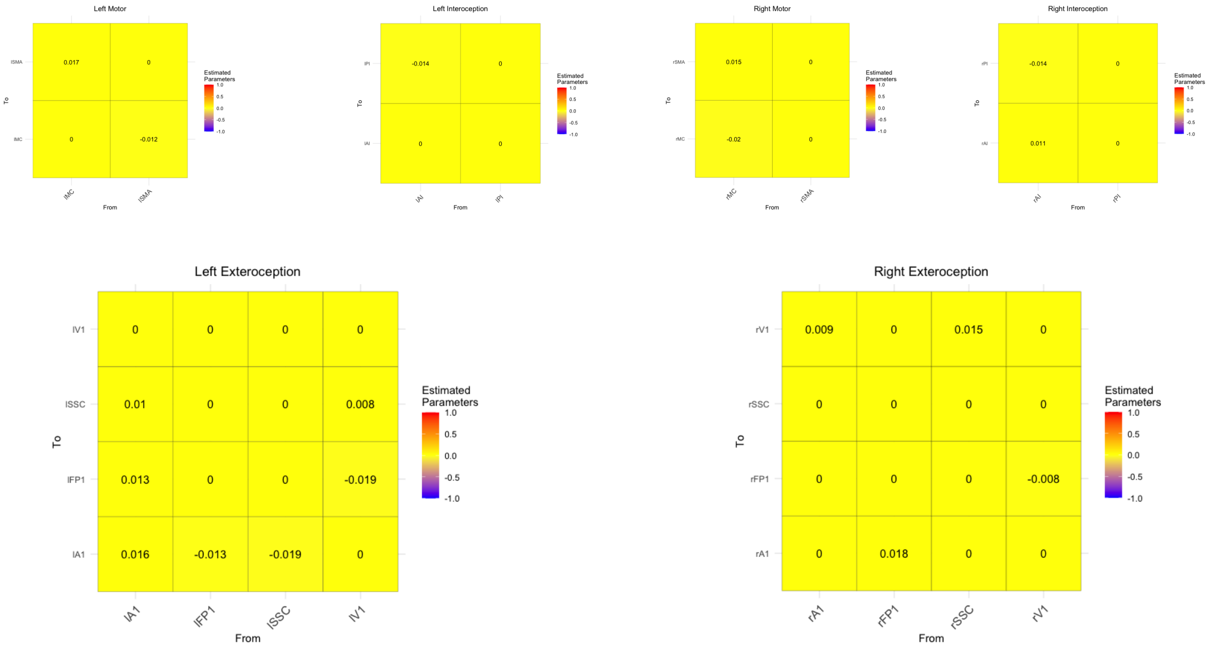

Supplementary Figure 1: Estimated parameters in the primary session

Group Mean Effective Connectivity

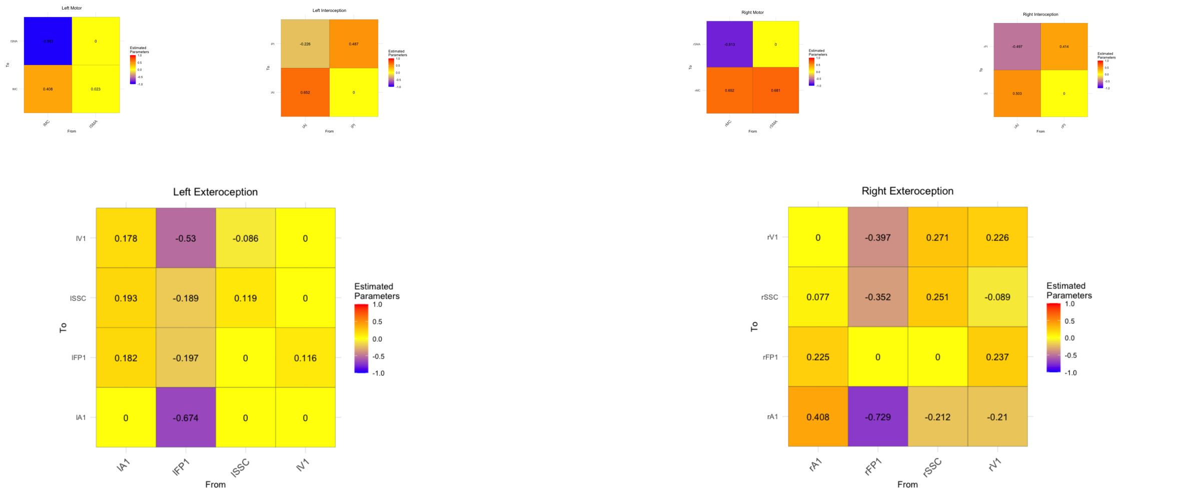

Changes in effective connectivity with BDI scores

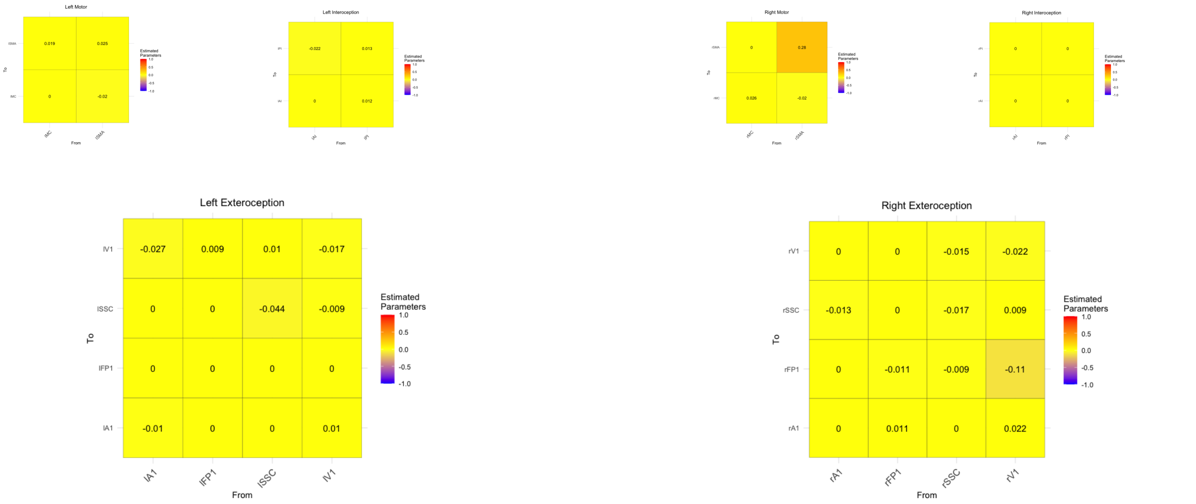

Changes in effective connectivity with treatment

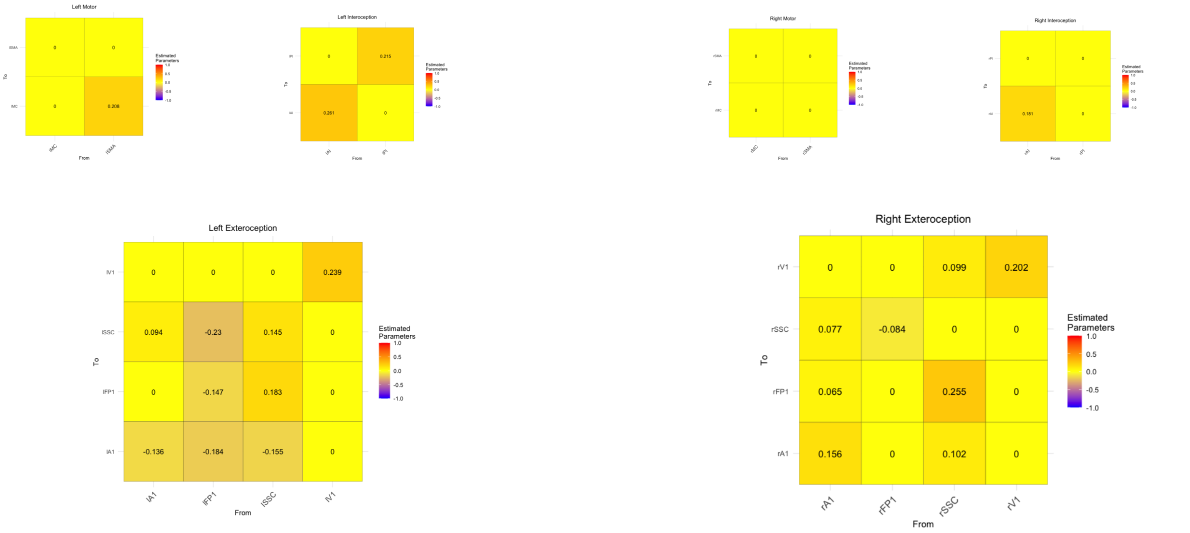

Supplementary Figure 2: Estimated parameters in the follow-up session
